# The enigma of weight: Figures, flux, and fitting in

**DOI:** 10.3389/fpsyg.2022.930360

**Published:** 2022-10-20

**Authors:** Katherine Wong, Maxine Myre, Nancy J. Moules, Danielle Lefebvre, Janelle M. Morhun, Jessica F. Saunders, Andrew Estefan, Shelly Russell-Mayhew

**Affiliations:** ^1^Faculty of Nursing, University of Calgary, Calgary, AB, Canada; ^2^Counselling Psychology, Werklund School of Education, University of Calgary, Calgary, AB, Canada; ^3^Psychology Convening Group, Ramapo College of New Jersey, Mahwah, NJ, United States

**Keywords:** weight, body image, body perception, bodily experience, experience of weight, weight change, philosophical hermeneutics, hermeneutic research

## Abstract

**Purpose:**

In Western society, the measurement of weight is prioritized over a person’s bodily experience. Hermeneutic philosopher Gadamer warned against the emphasis on measurement, rather than experience, in the medical sciences. An examination of the complexity of the experience of weight provides the opportunity to shift focus from quantifying the connection between health and weight to the experience of the person being weighed.

**Methods:**

This qualitative hermeneutic study aims to understand people’s experiences of weight from the interviews of professionals (*n* = 7) and lay experts (*n* = 10). Interviews were analyzed using an interpretive hermeneutic method.

**Results:**

The interviews revealed that weight was experienced as a number imbued with meaning and bias, as a number that could be manipulated, and as a constant and anticipated bodily change. Weight change was expected and often unwelcomed, despite weight being a quality of the body that is always in flux. External measures of weight meant to monitor wellness and health inadvertently became an unhealthy fixation that prevented some participants from fully participating in life events and appreciating the stages their bodies were in.

**Conclusion:**

Weight change is a necessary condition of being human, and bodies are and will be constantly changing. To achieve health and harmony, one must fit together the acceptance of change and their bodily experience of weight. It is often the preoccupation with weight, not weight itself, that gets in the way of living.

## Introduction

Being in the world necessarily involves weighing something: everybody, and every body, has a weight. However, in Western society (encompassing high-income, developed, and developing member countries of the Organization for Economic Co-operation and Development (OECD) in North America, Europe, New Zealand, and Australia), the meaning of the number on the weighing scale goes far beyond a body’s relationship with gravity. Weight also serves as an indicator of discipline, moral character, worthiness, and perhaps most prevalently, health (LeBesco, 2011). Weight-centric models of health are used in many professional fields, such as medicine and public health, as well as within public understandings of health (Bombak, 2014; Chrisler and Barney, 2017). While some weights are associated with a higher risk of health issues, the relationship between weight and health is complex and there is variability in what might be considered “healthy” for any particular individual (Wharton et al., 2020). Despite dominant beliefs, controlling one’s weight is not simple, yet many people find themselves attempting, sometimes obsessively, to do so. Even individuals with a weight in the so-called “healthy” range find themselves obsessing over their weight. The preoccupation with the number, and all the qualities and virtues associated with it, may present a larger problem for many individuals than the weight itself:

Health does not actually present itself to us. Of course, one can also attempt to establish standard values for health. But the attempt to impose these standard values on a healthy individual would only result in making that person ill. (Gadamer, 1993/1996, p. 107)

Hermeneutic philosopher Gadamer (1993/1996) emphasized the need to consider an individual’s experience of health as an indicator of wellness, rather than the measurement of vital signs (e.g., weight, blood pressure, and heart rate), as is practiced in modern medicine and science. Gadamer suggested that the objectification and quantification of bodily function and status, such as pain or wellness, requires a “violent estrangement from ourselves” (p. 70) to outwardly measure what is ultimately an inward awareness of one’s own body.

Health, as an embodied experience, is a state of effortless and unhindered engagement with the world that is only revealed when illness disrupts the ease of being in the world: the enigma of health is that health is only noticeable in its absence (Gadamer, 1993/1996; Aho, 2022). Merriam-Webster Dictionary (n.d.a) defined *enigma* as “something hard to understand or explain,” but in the case of health, the etymology of the word is more helpful. From the Latin root *aenigmae*, an enigma is something “which conceals a hidden meaning or known thing under obscure words or forms” (Online Etymology Dictionary, n.d.a). Health obscures itself: “It is only now, in its absence, that I notice what was previously there, or, more precisely, not *what* was previously there but *that* it was there” (Gadamer, 1993/1996, p. 74). When feeling unwell, one is forced to focus on new bodily challenges and sensations that shift one’s attention from being active in the world to the instability of occupying a now unhealthy body. The ideology of objectification that is prevalent in the natural sciences has seeped into the behaviors of individuals, as society and medicine have encouraged the hypervigilant self-monitoring of health domains (Mathiesen, 1997). This objectification moves individuals away from health as Gadamer (1993/1996) defined it: the “remarkable protected state in which we feel ourselves safely enfolded [in health] so that we are able, lightly and effortlessly, to embrace our desire for active participation in life” (p. 75).

Weight is one of the many facets of the human experience of health (e.g., pain or depression) that has become simplified and measured in an attempt to objectify a phenomenon that, as Gadamer (1993/1996) wrote, otherwise would not reveal itself. Weight, as an embodied experience, may also escape one’s notice unless it is revealed in the form of an external measurement (e.g., Body Mass Index, clothing size, or a number on a scale) or observation (e.g., reflection in the mirror or a photo on social media). Unfortunately, there are very few opportunities to escape the numerous external forces that shape and quantify one’s experience of their own bodily weight. Unlike health, weight is not something that can be hidden from others; as a characteristic of the physical body in which individuals present themselves, it is immediately noticeable. The instrumentation and quantification of weight, as well as the mass media’s constant reminder to “watch” one’s weight (Duncan, 1994), is so commonplace that the enigma of weight may present the opposite issue from Gadamer’s enigma of health: weight, even what might be considered a healthy weight, is conspicuous and front-of-mind for many individuals. If health is a state of unhindered and undisturbed engagement with the world, society’s fixation with the number on the scale is questionable as a health practice.

As with any method of measurement, there are limits to what quantification and objectivity can tell us about weight and how it is lived out in the world (Gadamer, 1993/1996). Stepping out of the weight-centric view of health and wellness provides the opportunity to pose different questions about weight as an embodied experience. As Gadamer (2007) wrote: “Thus, what is established by statistics seems to be a language of facts, but which questions these facts answer and which facts would begin to speak if other questions were asked are hermeneutical questions” (p. 84). As the practice, philosophy, and tradition of interpretation, hermeneutics provides an alternate approach to weight than those found in the natural and medical sciences, so that weight might be understood differently than simply a number on a scale (Gadamer, 1960/2004; Moules et al., 2015).

## Materials and methods

This article presents one of the findings (the enigma of weight) from a larger hermeneutic study led by the last author, guided by the research question: How might we understand the social complexities of weight? This study was underpinned by Gadamer’s philosophical hermeneutics, from which Moules et al. (2015) developed a research method aimed at deepening or changing the understanding of phenomena. As a practical, rather than theoretical, philosophy applied to research, hermeneutics is a particularly useful method to explore topics in practice disciplines, such as weight-related fields.

### Recruitment and sample

Hermeneutic research is informed by participants who can best speak to the topic under investigation; therefore, data collection in hermeneutic studies focuses on individuals who have experience with the phenomenon, as the topic is informed by their experiences, insights, and the meanings they attribute to it (Moules et al., 2015). Participants included: (a) expert professionals who work in weight-related fields (i.e., researchers, practitioners, and/or policymakers in psychology, psychiatry, counseling, and public health who are experts in weight), and (b) experts by experience, henceforth referred to as lay experts, who had or had not experienced weight issues, who could inform “common knowledge” understandings of weight. Both lay experts and professional participants had unique experiences and knowledge of weight, and the diverse perspectives served to deepen our understanding of the social complexities of weight as a topic. Professional participants were recruited through an international listserv of interdisciplinary professionals, 24 of whom were contacted by the listserv director. Of the 24 professionals contacted, nine responded to the participation request and seven agreed to participate. Professional participants had 10–37 years of experience as weight experts in the fields of psychology, psychiatry, counseling, and public health from the United States, Canada, Australia, and across Europe. All professional experts identified as female and Caucasian.

Lay experts were recruited through advertisements on the social media accounts of the last author’s lab and through snowball sampling. Lay experts identified their positions as students, physiotherapists, human resources workers, social workers, project managers, and teachers, and were between the ages of 21 and 54 years. Though there were no restrictions placed on geographic location, all lay experts were Canadian and identified as Caucasian or Asian and female.

### Data collection and analysis

Semi-structured interviews, 60–90 min in length, were conducted by telephone (*n* = 15) and in-person (*n* = 2). Interviews with professionals (*n* = 7) were conducted by the principal investigator, while interviews with lay experts (*n* = 10) were completed by two senior doctoral-level research assistants. Telephone interviews allowed the authors to broaden recruitment and not be limited to one location geographically. This was especially important for the professional experts, as they were located around the world. In alignment with hermeneutic philosophy, hermeneutic research interviews have a conversational tone and are structured by the topic of inquiry rather than a set list of questions, which allows the interviewer to respond to the unique insights of the participants (Moules et al., 2015). As such, each interview includes a unique line of questioning. All interviews were audio-recorded for verbatim transcription of the data. This study received approval from the institutional ethics board and all participants provided consent.

Data analysis, which is synonymous with interpretation in hermeneutic research, involves the careful reading and review of interview data (Moules et al., 2015). Interpretive memos were generated during transcript analysis and refined into initial interpretations that were brought to the research team and further expanded upon during the team’s conversations through “in-depth, rigorous, reflexive, and communal attention to the data” (Moules et al., 2015, p. 118). The results of hermeneutic studies are interpretations as informed by the thoughtful and meticulous work of the research team, the interview data, hermeneutic philosophy, relevant scholarly literature, and other literary sources that serve to deepen, transform, or reveal taken-for-granted facets of the topic that lead to an elevated or changed understanding (Moules et al., 2015). Hermeneutic inquiry does not require researchers to act as an objective observer of the topic under investigation: a hermeneutic researcher cannot remove their subjectivity and experiences from the work, as what one understands prior to inquiry provides the foundation for interpretive work and the evolution of new understanding through data analysis (Gadamer, 1960/2004; Moules et al., 2015). As such, the experiences of the research team can be included as data if it is relevant to interpretive generation.

In our exploration of the enigmatic quality of weight as it is experienced and understood by professionals and lay experts, several interpretations were derived from transcription analysis and team discussions. The focus of hermeneutic research is on the topic under investigation and not the participants’ individual experiences of that topic; thus, participants are not named individually as the aim is not to represent their stories, but to explicate what can be learned or understood of the topic itself (Moules et al., 2015). Quotations from participants are identified as coming from either a Lay Expert or Professional interview.

## Results

During the interviews, participants shared several experiences and beliefs regarding the weight that spoke to its enigmatic quality. Weight was a topic that consumed the minds of some participants, while for others, thoughts about weight hardly registered. Participants indicated that while weight is easily defined from an empirical mindset, weight as it is experienced and understood by both lay and professional experts was much more complex and multifaceted: satisfaction with one’s weight was not as simple as fitting in to a standardized health measure. Participants noted the way that bodies (i.e., figures) are spoken or thought of, and the figures of speech used to describe them in our everyday language, which helped inform our understanding of the topic. Weight changes, or weight in flux, that were already experienced or anticipated was another aspect of weight that helped shape our understanding. Some participants expressed that they first noticed fluctuations in their weight during life events when one would typically anticipate physical changes, such as puberty, attending post-secondary school for the first time, pregnancy, and menopause:

I definitely think that when everything was great and I had a fast metabolism and was super slim, weight was never really something that was on my mind until I started noticing [during university] that I could actually gain weight pretty easily. (Lay Expert)

The disconnect between the external body and the internal mind, everyday language, and anxiety surrounding weight changes during certain life stages or events all contributed to a situation that Gadamer (1993/1996) had warned against the fixation on an objective measure in place of an embodied experience. Weight is simply defined as “relative heaviness or mass” (Merriam-Webster Dictionary, n.d.b). presented as a number, but the enigma of weight comes into play when the social complexities and meaning that hide behind the number are revealed.

### Figures of speech (language and prejudice)

All this stuff [about weight] we take in and I don’t even know if we know how we’re taking it in. (Professional)

Participants held beliefs about weight that were informed by their prejudices, which in the hermeneutic sense of the word entails neither a positive nor negative meaning (Gadamer, 1960/2004).

One’s prejudices, or the “biases of our openness to the world” and the “conditions whereby we experience something” (Gadamer, 2007, p. 82), may influence how one perceives their weight as a number rather than as a bodily experience.

How one comes to understand what they think or do about their weight may be informed by familial or parental beliefs and behaviors, one’s culture, everyday language, personal experience, and standards set by science and medicine. The prejudices one “takes in” and internalizes about weight are influenced by the social context that one is born into, as there “is always a world already interpreted, already organized in its basic meaning relations” (Gadamer, 2007, p. 87); put another way, the influence of one’s social context cannot be underestimated in the formation of one’s understanding of the world. In Western society (though we recognize that this may be the case in other cultures and countries), fatness is viewed negatively, whereas thinness is valued and celebrated. These beliefs develop early in life and their origins and accuracy may never be questioned. There is quantitative and qualitative evidence to suggest that in the United States and Australia, children as young as 36 months (Ruffman et al., 2016) and 3 years old (Jennull et al., 2021) express preferences for observing slimmer-figured individuals and internalize the idea that larger bodies are less preferable to smaller ones.

[In] the setting that I grew up in, there weren’t many large people around. It became easy just to believe that those few that were, there was something wrong [with them]. All of these things you never quite leave behind. (Professional)

Some participants commented that their weight was not a preoccupation for them until it was brought to their attention by others. Participants recounted situations during their youth where well-meaning healthcare professionals and loved ones had made comments that focused their attention on weight, which continued to influence their thinking or behavior into adulthood:

My older brother went through a period right before he became a teenager where he got a tiny bit rounder, but not a lot. But I remember my dad kind of basically putting him on a diet. Sort of like, “No kid of mine is going to have a tiny bit of a belly.” I remember even then it was nothing really. But I guess my dad was worried it was going to somehow escalate or something. Meanwhile, my brother grows up, and weight is not an issue – but even in adulthood, I mean he’s in his fifties now, and I’ve seen him on multiple occasions criticize himself for having like a little bit of flesh. It became a weak spot for him, or a place, something that he feels critical of himself about. (Professional)

Widely held societal beliefs about weight may also influence professional practice. Several professional expert participants expressed frustrations with their own discipline’s approach to weight management:

Of course, when it comes down to it, the obesity prevention people are about ‘all I’ve got to offer is eat less and exercise more,’ even though it’s been shown not to work. You’re just banging on about stuff that doesn’t work; how can you call yourself a scientist? I get quite upset with those colleagues on a number of fronts. (Professional)

It may also be the case that one is unaware of or experiencing tensions with the prejudices they hold, which can result in situations where it is difficult to understand what one believes. One professional expert called for a more humane and non-judgmental approach to weight in their profession, stating *“There needs to be a lot more humanity…there’s a tendency to judge everybody in the same way,”* but then went on to say that individuals struggling with anorexia nervosa *“do look frightful…I’m seeing somebody and it’s like a skeleton, you know.”* Another professional expert discussed the tensions that arise when colleagues or weight specialists, who have built their careers on tackling the “obesity epidemic,” are confronted with an alternate understanding of weight or the upstream causes of obesity:

Once people had hung their career in specializing in ‘obesity,’ whether it’s treatment or prevention or something else, to come back to them and say “You invented this, and you’ve missed all these key aspects that you need to be taking into account, so that you’re misrepresenting what’s really happening in people’s lives. In fact, you’re making things worse for people.” That’s a bitter pill to take and will get a lot of resistance. (Professional)

Commonly held beliefs and seemingly innocent language about weight have the power to shape how individuals and society perceive changes in weight. Colloquial expressions establish certain expectations and mindsets around weight; it is not uncommon to see a magazine headline announcing that a celebrity “got their body back” following pregnancy, as if their body had ever left them. The phrase “maintaining your weight” suggests that weight is something that should not change, despite the fact that weight is inextricably tied to a body that *will* change with time.

### The divisive capacity of quantification

Gadamer (1993/1996) warned that objectification and quantification of embodied experiences creates a separation between the mind and body, undermining how an individual feels in their body in favor of external tools of measurement that have been validated by the scientific community and appear to offer the certainty or mastery of knowledge about the human body. While there are many positive health outcomes that result from advancements in science, it is important to remember that, as Gadamer (1993/1996) stated,

…we can take up an external view on the world and observe among all its various phenomena our own bodily experience. Through its methodological procedures modern science has succeeded in objectifying this experience… We would then have to ask: what is the relationship between science and bodily experience? (p. 71)

An individual can internalize and interpret an external measurement of weight, a number that one lay expert participant stated, *“is deceptively easy”* but *“imbued with a lot of meaning”* and that guides future actions, behaviors, thoughts, and decisions. Yet the control over what calories go in and what calories are expended does not necessarily lead to successful weight loss or maintenance. Relying on objective measurements alone ignores an important source of knowledge that can be gleaned from engaging with the internal sensations, cues, and experiences of the person being weighed.

According to self-reported weight, intuitive eating is linked with weight stability and dieting is not. You know, dieting is not linked to weight stability, it’s often linked to weight gain. (Professional)

Participants acknowledged that while the scale can tell us one aspect of the broader picture of health and wellness regarding weight, the body should not be neglected as an equally powerful indicator of a person’s needs and the appropriate behaviors and interventions to explore. The expectation that those who occupy larger bodies listen to outward indicators, such as Body Mass Index or weight, to dictate behaviors may be less helpful than listening to their bodies:

For certain individuals, they’re allowed to eat intuitively. That is not disruptive for them, even the thin people or, you know, people even at the middle of the weight spectrum they’re left alone. They can eat intuitively. However, due to weight stigma people at the high end of the weight spectrum, they’re socially expected to diet. They’re expected to ignore their hunger cues. (Professional)

Monitoring weight provides the illusion of control and, like many forms of measurement that are predominant in the natural sciences, may appear more powerful than paying attention to the embodied experience of the construct being measured. Weight is a physical aspect of embodied life that many wish to control, maintain, and manipulate. However, “the human being does not exist as an objectively present substance with measurable properties” (Aho, 2022, p. 178), and there is always more at play in the make-up of a body than what can be simply measured. Weight is not a constant variable in the lifespan of a human being and is not so easily maintained or controlled, despite predominant beliefs that weight is something that can be manipulated with diet, exercise, and discipline:

How do I conceive of weight? It seems to be this thing that people think is very manipulatable, that I don’t think is quite as manipulatable as people seem to think. (Professional)

### Weight in flux

I remember [a high school friend] telling me “My thighs started touching.” I was like “Not a big deal, that happens” …that’s just normal development. (Professional)

As weight is inextricably tied to the body, it is unavoidable that as the body changes, weight changes will follow; however, weight fluctuations are often more conspicuous than fluctuations in someone’s life. Not all weight loss is a step in a positive direction (e.g., weight loss due to illness) and not all weight gain indicates a trend toward unhealthy habits (e.g., overcoming an eating disorder, pregnancy, treatment for illness, medication side effects, increased muscle mass, or increased height). Natural, and often anticipated, physical changes in the body and weight, like those that occur during puberty or pregnancy, typically coincide with fundamental shifts in life stages and roles. Many participants discussed their experiences of weight in relation to life-stage milestones, such as losing weight for a wedding, gaining the “Freshman 15” in university, or the changes that occur in a woman’s body during pregnancy.

Menopause happens and your body is just sort of different. (Professional)

Even if your weight stays the same as you age, it’s just like your body is just ‘Oh, everything sags,’ you know. (Professional)

We know that lots of pregnancies do alter them [bodies], you know, when women gain a lot of weight (Professional)

One of our co-authors shared a story about her friend who had recently become a mother.

Immediately after her cesarean section, the anesthetist stated, “your body already looks ready to go to the beach,” as if her body had not transformed to grow a human, as if after such a fundamental shift from being responsible for oneself to being a mother would not require some kind of fluctuation. One professional participant expressed their desire to continually develop their professional knowledge, stating that “*there’s nothing to look forward to becoming fossilized and fixed.”* It is interesting that in Western society, one is expected, and often encouraged, to change and grow mentally. The desire to evolve thinking does not seem to extend to the evolution of the body:

we can change our minds, but our bodies need to be “ready for the beach” as soon as the baby is born. The data did not indicate that participants felt there was anything to look forward to in the physical changes that a body undergoes as it moves through life.

German philosopher Martin Heidegger wrote, “we do not listen because we have ears. We have ears, we are endowed with bodily ears, because we listen” (Heidegger, 1978, as cited in Chretien, 2015, p. 107). French philosopher Jean-Paul Sartre wrote, “we say that a man is a sexual being because he has sexual organs. What if the opposite were true? What if he possessed sexual organs only because he is fundamentally and radically sexual?” (Sartre, 1965, as cited in Chretien, 2015, p. 107). Perhaps we have fluctuating bodies that undergo weight changes that we must adapt to (e.g., try to lose weight, change our mindset about our weight), or perhaps we have adaptable bodies precisely because we are “fundamentally and radically” fluctuating beings, in both mind and body.

The Ancient Greek philosopher Heraclitus and his River Fragments writings on change help to elucidate how one might interpret weight as an aspect of life that is always in flux, rather than as a variable that can be controlled and manipulated. Western metaphysics has at its core the Paramedian belief in perfection as an unchanging, static being (George, personal communication, 2020), and Western standards for health and beauty share these sentiments. In contrast to

Parmenides, Heraclitus believed that all beings exit in a state of constant change (Graham, 2019; George, personal communication, 2020; Kirk, 1951). Heraclitus’ River Fragments pose a hermeneutical problem, as only segments of his writings have survived antiquity. Still, they have been interpreted and reinterpreted across many years and by later Greek philosophers. Kirk (1951) and Graham (2019) agreed that Fragment B12 is likely the interpretation that most authentically reflects Heraclitus’ meaning: “On those stepping into rivers staying the same, other and other waters flow.” Put another way, a river only remains a river by changing what it contains. Graham (2019) explained:

… some things stay the same only by changing. One kind of long-lasting material reality exists by virtue of constant turnover in its constituent matter. Here constancy and change are not opposed but inextricably connected. A human body could be understood in precisely the same way, as living and continuing by virtue of constant metabolism…Heraclitus believes in flux, but not as a destructive constancy; rather it is, paradoxically, a necessary condition of constancy…Heraclitus’ flux doctrine is a special case of the unity of opposites, pointing to ways things are both the same and not the same over time. (Graham, 2019, pp. 10-11)

Kirk (1951) also emphasized Heraclitus’ flux as it pertains to the body: “Man, it is true, is in unceasing change: he is constantly growing older and as he does so the structure of his body alters” (Kirk, 1951, p. 40). The living body is in a state of constant flux, all the way down to cellular activity. Fat cells (found in adipose tissue) are not exempt from this. The only bodies in stasis, “fixed and fossilized,” are dead ones (Bishop, 2011).

Participants offered their reflections on changes in their weight, how they perceived themselves at different weights, and how their life situations related to the number on the scale. One lay expert participant reflected on how she compared her weight during a natural period of change

(puberty) to that of her parents’ weight and stated,

It could also have been my parents’ weight. It wasn’t fluctuating the way mine was as I was growing up and undergoing various changes related to puberty. So, maybe the constant visual of themselves and their health status and also their exercise and diet regimes was something I didn’t relate to as much, because there was so much more flux in my life. (Lay Expert)

The changes that participants described were not only physical, but also encompassing how their thinking around their weight and bodies evolved:

There were ebbs and flows in how I looked and perceived myself. (Lay Expert)

Another participant shared the need to adjust her mindset following physical changes to her body:

I think there’s going to be periods of shock on my body, and I think there’s going to be periods of time where I kind of need to readjust and learn more about how my body has changed and learn how to cope with it in a way that’s healthy. (Lay Expert)

While each participant’s experience of weight change may have led them to different conclusions, actions, or mindsets, change itself was anticipated, though perhaps not welcomed and accepted. The interpretation of weight as in flux might facilitate the acceptance of fluctuations in weight without the burden of anxiety and negative body image that seemed to accompany those changes. As Kirk (1951) emphasized, “The river fragments, then, seem to exemplify not the constancy of change… but the regularity of natural change in one particular manifestation [e.g., a body]” (p. 37). Of course, there are instances where changes in weight may signal that something is amiss (e.g., hormonal imbalances or disordered eating). Yet, in many cases, even those who are considered to have a “healthy” weight still obsess over the number on the scale, rather than accept that change will, and must, occur. If health is a state of being that largely goes unnoticed to the individual who is healthy, it is the preoccupation with weight, more so than the number, that may signify and result in unhealthiness.

## Discussion

Gadamer (1993/1996) reframed health as a state of unhindered participation in life, where one is able, “lightly and effortlessly,” to engage in the world (p. 75). However, there was nothing light or effortless about the way participants described their experiences of weight. Even individuals who were happy with their weight at the time of their interview dreaded periods of life where they anticipated their weight would change. For some participants in this study, weight was more than a physical burden, but a preoccupation that hindered their full participation in the world:

I’ve always put an emphasis on my weight, on what I ate. The [my] kids saw it continuously. They saw that I would yo-yo diet. My weight would go up and down. …There were times…while my kids were growing up, they saw that sometimes mom would have her own meal. …They saw me going to the gym, every day, continuously, forever. (Lay Expert)

### Not weighed down

Concerns and practices related to weight—gains, losses, fluctuations, or maintenance—may appear healthy in conventional biomedical models of health; however, when one considers.

Gadamer’s insights of health, as something that remains unnoticed until it is replaced by illness, the extent to which we think about weight is unhealthy. Bodies cannot return to what they were 10 years ago, nor to their pre-pregnancy or pre-menopausal conditions. Bodies and weight change and achieve a new balance in their altered state: “The life of the body always seems… to be something which is experienced as a constant movement between the loss of equilibrium and the search for a new point of stability” (Gadamer, 1993/1996, p. 78).

Weight-centric approaches to health that privilege measurement over experience have historically dominated how we understand and educate about weight. Norms and values about weight communicate how to judge and modify our bodies to conform to an elusive and ever-changing standard. We learn from an early age what type of body is seen as valued and “healthy,” or at least acceptable, and what body types that require intervention and modifications to reach societal acceptance. Babies are weighed the minute they are born and at almost every subsequent health visit in medial contexts. In educational settings, fitting in becomes literal through desks and gym uniforms. We learn about weight directly and indirectly as something that needs to be managed and controlled or at the very least, closely monitored and evaluated. However, we do not learn how to live in and honor our bodies.

Gadamer (1993/1996) offered another helpful reminder from Heraclitus: “Among the fragments of Heraclitus, we find this profound observation: ‘the concealed harmony is mightier than the revealed.’” (pp. 75–76). To be in harmony, as Gadamer and Heraclitus suggest, means to accept that in order to live, our body weight must change. At the same time, it means to let go of the preoccupation of changing our weight; we need to stop investing time, energy, and resources into some static magical number that does not exist. The word “harmony” has etymological roots in the word *harmos*, meaning “to fit together” (Online Etymology Dictionary, n.d.b). Harmony in the context of weight and changing bodies is not a reflection of fitting in to societal expectations for weight, but a fitting together of our acceptance of change and our bodily experience. Relying on objective and external measures of weight neglects to consider one’s bodily experience of being in the world. For some participants, the preoccupation with weight was an unhealthy fixation, which drew attention away from major life events like motherhood, marriage, and menopause. Preoccupation with weight is an unhealthy obsession. Weight change is a necessary condition of being human, and our bodies are and will be constantly changing. We grow, we develop, and we move through stages; all of these require that our bodily weight changes over time. The authors recognize that not all individuals are preoccupied with their weight or place the same emphasis on standardized measures to determine their weight satisfaction, and that some individuals do experience health concerns related to their weight. However, our study findings suggest that weight itself is not always a good proxy for health or harmony and our preoccupation with weight moves us away from both. It is our preoccupation with weight, not weight itself, that gets in the way of living.

## Limitations and methodological considerations

This study included professional participants from Canada, the United States, Australia, and countries across Europe (OECD countries), who all identified as female and Caucasian, and had 10–37 years of experience in weight-related fields. All lay expert participants were from Canada who identified as Caucasian or Asian and female. As such, this study lacks a diversity of perspectives from other genders, cultures, and ethnicities. The small sample size of this study is methodologically consistent with hermeneutic research (Moules et al., 2015), and generalization is not possible. However, it should be noted that generalization is not the objective of hermeneutic research; rather, high-quality hermeneutic research should open up a topic to be understood in new ways, allowing research consumers to consider topics that arise in their practice with novel thinking or understanding, which may lead to changed behaviors (Moules et al., 2015). A small sample size provides the ability to conduct an in-depth interpretive analysis that allows the unique contributions of participants, along with relevant literature and research, to extend our understanding of the topic of inquiry. Replication is not possible due to the interpretive quality and the style of interviewing that is consistent with hermeneutic research methodology (Moules et al., 2015).

## Data availability statement

The original contributions presented in the study are included in the article/supplementary material, further inquiries can be directed to the corresponding author.

## Ethics statement

The studies involving human participants were reviewed and approved by the University of Calgary Conjoint Research Ethics Board (REB18-0684). The patients/participants provided their written informed consent to participate in this study.

## Author contributions

SR-M (primary investigator), NJM, and AE contributed to the conception and design of the study. SR-M, KW, MM, DL, JM, and JFS performed the initial qualitative analysis. KW wrote the first draft of the article with the assistance of MM. SR-M and NJM reviewed the initial draft of the article. All authors contributed to the final analysis of the data, manuscript revisions, and approved the submitted version.

## Funding

This study is supported by the Social Sciences and Humanities Research Council of Canada, under Grant 435–2018-0116.

## Conflict of interest

The authors declare that the research was conducted in the absence of any commercial or financial relationships that could be construed as a potential conflict of interest.

## Publisher’s note

All claims expressed in this article are solely those of the authors and do not necessarily represent those of their affiliated organizations, or those of the publisher, the editors and the reviewers. Any product that may be evaluated in this article, or claim that may be made by its manufacturer, is not guaranteed or endorsed by the publisher.
